# Computer Vision System for Mango Fruit Defect Detection Using Deep Convolutional Neural Network

**DOI:** 10.3390/foods11213483

**Published:** 2022-11-02

**Authors:** R. Nithya, B. Santhi, R. Manikandan, Masoumeh Rahimi, Amir H. Gandomi

**Affiliations:** 1 School of Computing, SASTRA Deemed University, Thanjavur 613401, India; 2 Faculty of Electrical and Computer Engineering, Shahr-e-Rey Branch, Islamic Azad University, Tehran 1815163111, Iran; 3 Faculty of Engineering and Information Systems, University of Technology Sydney, Sydney, NSW 2007, Australia

**Keywords:** fruit defect detection, machine learning, deep learning, convolutional neural network, mango

## Abstract

Machine learning techniques play a significant role in agricultural applications for computerized grading and quality evaluation of fruits. In the agricultural domain, automation improves the quality, productivity, and economic growth of a country. The quality grading of fruits is an essential measure in the export market, especially defect detection of a fruit’s surface. This is especially pertinent for mangoes, which are highly popular in India. However, the manual grading of mango is a time-consuming, inconsistent, and subjective process. Therefore, a computer-assisted grading system has been developed for defect detection in mangoes. Recently, machine learning techniques, such as the deep learning method, have been used to achieve efficient classification results in digital image classification. Specifically, the convolution neural network (CNN) is a deep learning technique that is employed for automated defect detection in mangoes. This study proposes a computer-vision system, which employs CNN, for the classification of quality mangoes. After training and testing the system using a publicly available mango database, the experimental results show that the proposed method acquired an accuracy of 98%.

## 1. Introduction

Mango is a major fruit crop in India that is rich in vitamins and minerals [1]. The total production of mangoes is estimated to be 50.6 million tons worldwide, 39% of which occurs in India. Thailand and China are the next highest producers. Uttar Pradesh, Tamil Nadu, Telangana, Andhra Pradesh, Kerala, Bihar, and Karnataka are the main producers in India [2]. While mango production is increasing every year in India, the total export of mangoes from India is very low due to the lack of nondestructive, quality and reliable automated tools and techniques. The quality of mangoes can be identified by their skin, which displays green spots and yellowish speckles. In India, quality grading of mango fruit is done manually by experienced evaluators, which is based on defect detection of the fruit’s surface. Manual sorting involves workers having to perform sensory tasks in a large capacity and for long working hours. Thus, the manual grading of mango fruit is a time-consuming process [3,4,5]. In addition, this process requires a significant number of employees, which increases the cost of production and can lead to uncertainty and inaccurate results because individual judgments are subjective and inconsistent across fruit objects. Overcoming these limitations requires the help of computer image sensors to be more effective and efficient. Therefore, we propose a reliable method for automatic defect detection of mangoes and a computer vision system for automated grading.

The defect detection of mango fruits using a computer vision system includes three macro steps: preprocessing of an input image, feature extraction, and classification of the input image. The computer vision system in the agricultural domain enhances the quality of food products for the export market, in which fruit grading is an essential process to select quality fruits. Hence, there is a need for an intelligent computer vision system for fruit grading. Over the past few years, non-destructive machine learning techniques have been employed for efficient fruit quality assurance and have become an integral part of developing computer vision systems for fruit defect detection [6,7]. In addition, image processing and data mining techniques are widely employed in the agricultural domain for computerized defect detection and quality grading of produce. The objective of this work was to develop a computer vision system for mango defect detection using advanced machine learning techniques, such as the convolutional neural network (CNN).

The remainder of the paper is organized as follows. A summary of the related works is described in Section 2. In Section 3, the outline of the proposed model and methodology are explained. Section 4 presents the performance measures to assess the effectiveness of the proposed methodology. Section 5 discusses the experimental results, and Section 6 provides a comparative analysis of the proposed method and existing works. Section 7 contains concluding remarks about the proposed methodology and obtained results.

## 2. Related Work

Machine learning and image processing techniques have been extensively used in the agricultural domain over the last few years. In particular, computer vision systems provide a nondestructive, low-cost, fast and reliable means for fruit defect detection. Thus far, several works have investigated automated fruit defect detection based on the fruit’s surface.

Patel et al. [8] proposed a computer vision system for the non-destructive physical characterization of mangoes considering various morphological features and multilinear regression models for quality grading and obtained an accuracy of 97.9%. Similarly, Patel et al. [9] developed a computer vision system for defect detection of mangoes using a reflected ultraviolet imaging technique. They found that a band-pass filter of 400 nm is appropriate to detect the defective mangoes which were not identified by the RGB color camera. Nandi et al. [10] introduced a computer vision methodology for grading mangoes based on maturity and quality. They used fuzzy incremental learning for grading mangoes and obtained an accuracy of 87%. Nandi et al. [11] presented a machine vision system for the prediction of mango maturity level. After various texture features were extracted from mango images, the relevant features were selected by the recursive feature elimination technique using SVM as a classifier. Huang et al. [12] proposed a computer vision methodology for the non-destructive detection of mango quality, which includes a colorimetric sensor array, principal component analysis, and support vector classification for qualitative discrimination. They classified the mangoes into three grades and obtained an accuracy of 97.5%. Guojinet et al. [13] introduced a computer vision technique for mango appearance rank classification based on appearance characteristics using extreme learning machine neural network to rank the mangoes. Sahuet et al. [14] developed an automated tool for maturity and defect identification of mango fruits using digital image analysis considering size, color, and shape features of the mangoes. Andrushia et al. [15] presented an automatic skin disease identification system for mangoes in which extracted features such as texture, color, and shape are selected from a digital image using the artificial bee colony optimization and SVM as a classifier. Momin et al. [16] developed a computer vision system for grading mango fruits based on geometry and shape features using image processing techniques such as global thresholding, color binarization, median filter, and morphological processing and achieved an accuracy of 97%.

Additionally, Ragavendra et al. [17] proposed an optimal wavelength selection methodology for mango defect detection and obtained an accuracy of 84.5%. Kumari et al. [18] developed an automated system for mango defect detection using enhanced fuzzy k-means clustering, maximally correlated principal component analysis, and back propagation-based discriminant classifier. Patel et al. [19] presented a monochrome computer vision system for mango defect detection, which achieved an accuracy of 97.88%.

## 3. Methodology

**Figure 1:**
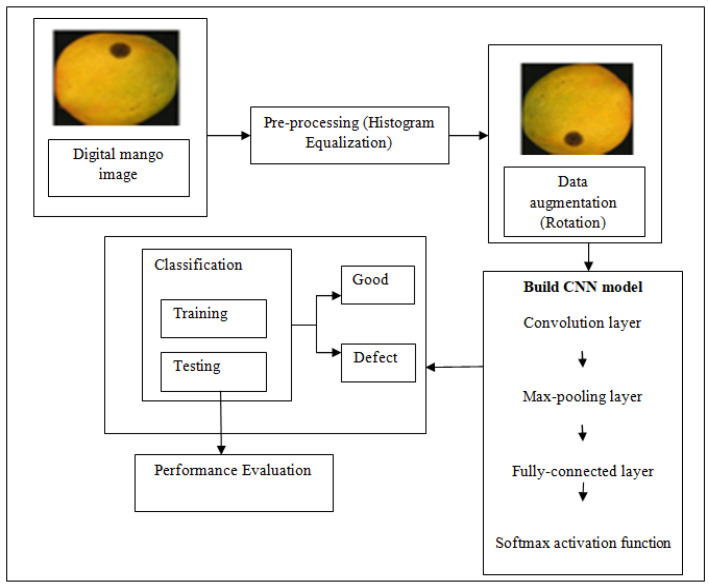
Overview of the proposed methodology.

The proposed CAD system includes the following phases: (a) preprocessing to enhance the image quality; (b) data augmentation to increase the data samples, and (c) classification of mango images as either good or defective. The outline of the proposed methods is shown in Figure 1.

### 3.1. Dataset

The dataset used in this study contains 50 good and 50 defective Kent mango images 1024 × 1024 pixels in size. This database is publically available on the following website: http://www.cofilab.com/portfolio/mangoesdb/ and accessed on 1 March 2022.

### 3.2. Preprocessing

Preprocessing is an important step in the computer vision system that is performed to enhance image quality. Noise removal and image enhancement to show defective areas on the surface of fruits are preprocessing techniques considered in this work.

#### 3.2.1. Histogram Equalization

Histogram equalization (HE) is a widely used preprocessing technique to improve the quality of the digital image. This technique is useful to improve the contrast of the image with the histogram of the image. HE works by uniformly distributing the pixel intensity values [20] which is accomplished by effectively distributing the most frequent pixel values. This approach is suitable for images with foregrounds and backgrounds that are both dark and bright. Let I be a given image represented as an m × n matrix of integer pixel intensities ranging from 0 to *L* − 1. L is the number of grey levels in the image, often 256. Let *p* denote the normalized histogram of *I* with a bin for each possible intensity.

$$p_{g}=\frac{number\;of\;pixels\;with\;intensity\;\left(g\right)}{total\;number\;of\;pixels}\;g=0,1,2,\ldots ,\;L-1.$$

The histogram equalized image *I*1 is represented by

$$I1_{\left(i,j\right)}=\left(L-1\right)\sum_{g=0}^{I_{i,j}}p_{g}.$$

#### 3.2.2. Adaptive Wiener Filter

A Linear filter like Adaptive Wiener Filter is useful for de-noising and smoothing. This filter reduces the mean squared error. The amount of smoothing performed by this filter depends on the local image variance. For the large variance, less smoothing is performed, and for the small variance, more smoothing is performed. This low-pass filter was applied in a local neighborhood of 3 × 3 pixel blocks of the image. The adaptive Wiener filter reduces the background noise without blurring, retaining edges high frequency and edge parts of an image.

### 3.3. Data Augmentation

**Figure 2:**
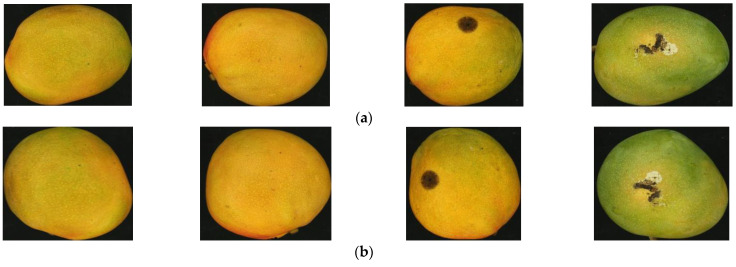

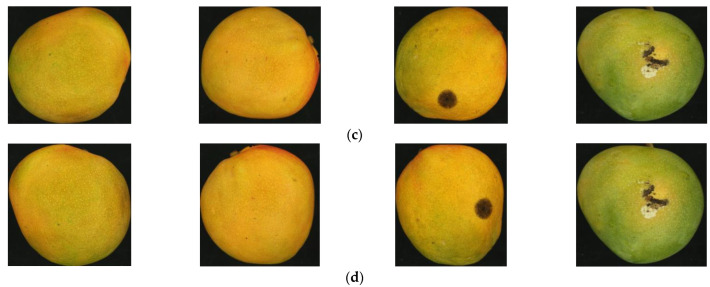
Images of mango samples: (**a**) Sample mango images; (**b**) Images rotated at 90°; (**c**) Images rotated at 180°; (**d**) Images rotated at 270°.

The data augmentation approach is widely employed in deep learning models to enlarge the data samples [21], which includes transformation techniques like rotation, flipping, shearing, and cropping. This is an essential process in deep learning models such as CNN that require a large number of data samples for training. This technique helps the deep learning model to enhance the classification performance by generalizing better and thereby reducing overfitting. While the database consists of 100 images of Kent mangoes (50 good and 50 defective) as mentioned, more images are needed to train the deep learning model. Therefore, scaling and rotation transformation were employed to augment the data samples. Through data augmentation, the initial 100 mango images were augmented into 800 images by applying rotation (90°, 180°, 270°, and 360°), flipping and scaling transformations. The sample images of the data augmentation process are shown in Figure 2.

### 3.4. Convolutional Neural Network

The convolutional neural network (CNN) is an improved artificial neural network [22] that is capable of classifying and recognizing defect regions in mango images via computer vision system. This neural network works by processing visual inputs and performing tasks such as object recognition, segmentation, and classification of images [23]. CNN is similar to a multilayer perceptron neural network.

#### 3.4.1. Convolution Layer

In CNN, the convolution layer applies a filter to obtain features from the input image and produce feature maps, or activation maps, as the output. The parameters used in convolution operation are the filter size F and stride S. The first convolution layer produces low-level feature maps such as edges, corners, and lines, while the subsequent layers generate high-level feature maps. The input image of size W × H × C (width, height, channels) is convolved with N kernel size of k × k × D, where D is the number of RGB channels and k is less than the image dimensions [24]. The convolution operations with N kernels generate N features. The convolution operations start from the top-left corner of the image and are repeated until the kernel reaches the bottom-right corner.

#### 3.4.2. Pooling Layer

The pooling layer, also known as the down sampling layer, reduces feature maps and the computational complexity of the CNN. This pooling operation is applied after a convolution by performing spatial invariance. The pooling operation minimizes the dimension of each feature map while preserving the most important features. The most common approaches employed in pooling operations are average pooling and max-pooling. In average pooling, the average of the values in the region of the feature map covered by the filter is used, whereas max-pooling takes the maximum value from the region of the feature map covered by the filter. In this study, max-pooling was employed.

#### 3.4.3. Fully Connected Layer

This layer uses the feature obtained from the pooling layer for the classification of input images. The fully connected layer flattened out the pooling output to a large vector. The last fully connected layer uses a softmax activation function to compute an output from 0 to 1 for each of the input images to predict. One or more fully connected layers are at the end of the CNN architecture.

#### 3.4.4. Rectified Linear Units (ReLU)

CNN applies a ReLU activation function to the convolved feature after every convolution operation in order to introduce nonlinearity into the model. ReLU is an activation function that is used to improve the training of deep convolutional neural networks. The advantage of the ReLU activation function is faster training [25].

#### 3.4.5. CNN Architecture

**Figure 3:**
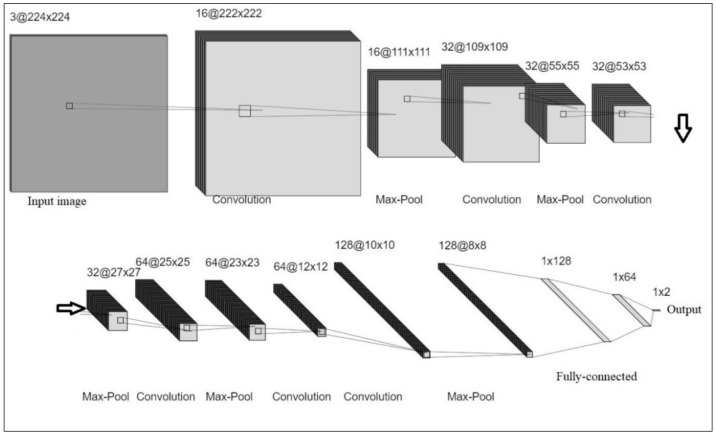
The proposed CNN architecture model.

Herein, a CNN architecture with 13 layers, namely 6 convolutions, 5 pooling, and 2 fully connected layers, was developed to perform automatic defect detection of mangoes without feature extraction and selection processes. The CNN operations, including convolution, non-linearity, pooling or sub-sampling, and classification, were performed on the input images that can be represented as a matrix with pixel values. Each convolution layer (1, 3, 5, 7, 9 and 10) is convolved with their respective kernel size (3, 4 and 8). A max-pooling operation is applied to the feature maps after the convolution operation. The final layer is the fully connected layer, which predicts the output. As seen in Figure 3 and Table 1, the input image of size is fed into the CNN, and then the input image pixel values are normalized from [0, 255] to [0, 1]. The convolution layer performs the convolution operation with kernel of size (3, 4 and 8), followed by a pooling operation, and then another convolution layer.

## 4. Performance Measures

A classification test and k-fold cross-validation were performed to determine the efficiency of the proposed method at identifying mangoes as either good or defective. In k-fold cross-validation, all images were utilized to train and test the proposed model, whereby all data samples were randomly divided into k groups [26]. One-fold was used for testing, and other k − 1 folds were employed for training. This process is repeated for other k − 1 folds. In this work, 10-fold cross-validation is used. In the classification test, the presence of a defective mango is positive, whereas absence of a defect is negative. Four different outcomes were possible as described in the confusion matrix in Table 2: True Negative (TN), True Positive (TP), False Negative (FN), and False Positive (FP) [27]. The classification performance was estimated by various measures, including area under the ROC (Receiver Operating Characteristic) curve (AUC), accuracy, sensitivity, and specificity based on the four possible outcomes, which are calculated as follows:

- TP—Mango images are classified as defective.
- FP—Mango images are misclassified as good.
- TN—Mango images are classified as good.
- FN—Mango images are misclassified as defective.

Accuracy = (TP + TN)/(TP + FP + TN + FN)

Sensitivity = TP/(TP + FN)

Specificity = TN/(TN + FP)

## 5. Experimental Results

**Figure 4:**
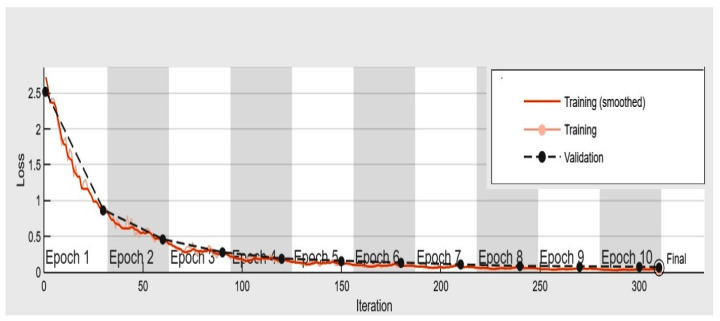
Training loss vs. validation loss.

In this work, we employed a deep learning method and binary classification to identify defective mangoes using a proposed computer vision system. Images of Kent mangoes were obtained from a publically available web database. The size of the Kent mango dataset images is 1028 × 1028 pixels. The data augmentation technique was applied to the database to artificially increase the number of mango images from 100 to 800 images which were used for the performance evaluation of the proposed system. In this system, human intervention is not required to obtain features from the input images. Section 5: The proposed CNN model is implemented in MATLAB and a computer with processor @2.83 GHz and 8 GB RAM. The proposed CNN model was trained with an Adam optimizer, 10 epochs and a different learning rate of 0.1, 0.01, 0.001, and 0.0001. The highest classification accuracy was obtained with a learning rate of 0.001 and a batch size of 32. The preprocessing technique, namely histogram equalization, was used to enhance the input images. Standard performance measures such as accuracy, recall, and precision were determined and used to assess the proposed system, and 10-fold cross-validation was employed for evaluation. An optimal fit is the objective of the proposed deep learning model that exists between an underfit and overfit model. An optimal fit is identified by a training and validation loss which is depicted in Figure 4.

**Figure 5:**
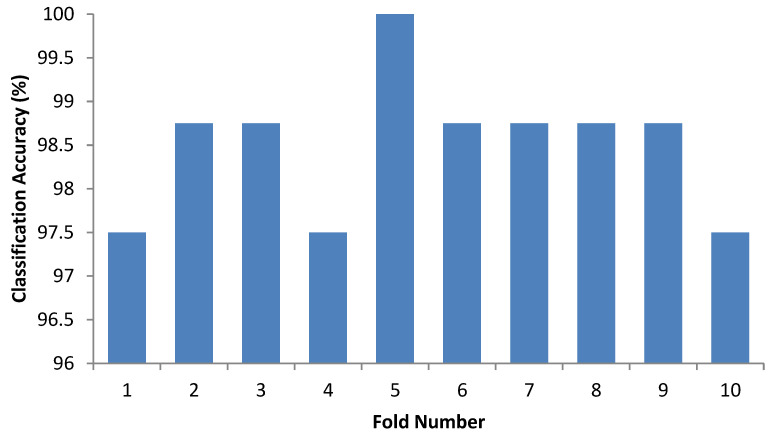
The classification accuracy of the 10 folds of cross-validation.

**Figure 6:**
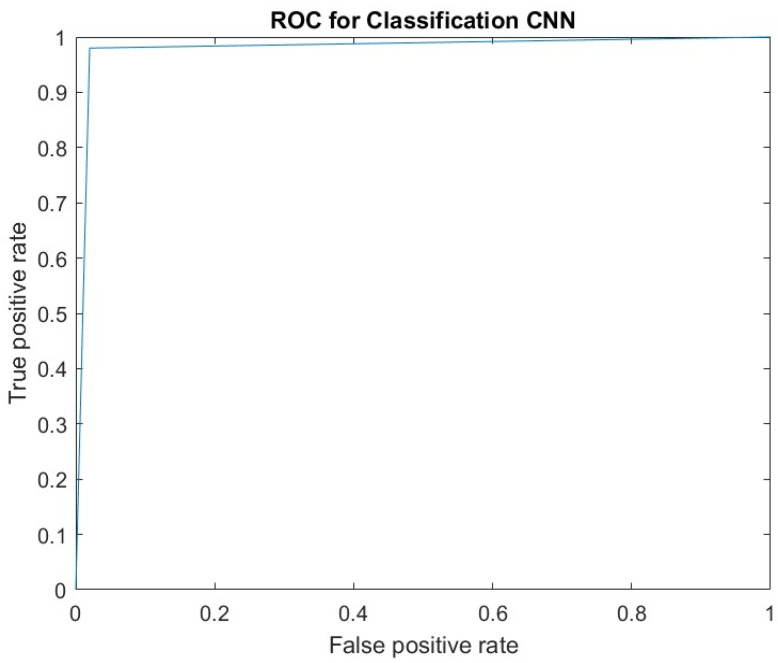
ROC curve for mango defect detection using the proposed CNN model.

The confusion matrix of 10-fold cross-validation for Kent mango defect detection is shown in Table 2. In this work, a positive value indicates defective mangoes, whereas good quality mangoes are considered negative. The classification performance measures (sensitivity, accuracy and specificity) of the proposed model in Table 2 reveal that less than 3% of the mangoes were misclassified in all folds. The average accuracy of the proposed model was 98.5%, suggesting that it can efficiently identify defective mangoes. Figure 5 presents the classification results of 10-folds. Figure 6 shows the ROC curve of the proposed method, which describes the classification ability of the binary classifiers and the obtained AUC value of 0.98.

## 6. Discussion

The proposed computer vision system is a simple and efficient tool for the automated detection of defective mangoes using advanced machine learning techniques. Experiments were carried out on a dataset of 800 mangoes. Because CNN requires a maximum number of images for better classification, data augmentation was performed to increase the number of data samples. In addition, the proposed model produced consistent results for all iterations. Table 3 summarizes the various approaches proposed by the researchers for automated defect detection of mango, including image processing techniques. Compared to related works, the classification result of the proposed deep learning model obtained the highest classification accuracy. As seen in the confusion matrix, the proposed CNN model correctly classified the good quality and defective mangoes.

Moreover, classification on the Kent mango database also demonstrates that the proposed model obtained good classification accuracy. One of the most important advantages of the proposed deep learning method is that, unlike the traditional machine learning model, it does not need segmentation, feature extraction, or selection processes. However, a disadvantage of the proposed deep learning model is that the training is computationally expensive and requires a large amount of data. The small dataset is one of the major challenges to train the deep learning model. Therefore, we applied data augmentation to obtain a larger dataset.

Training the deep learning model is essential to increasing its classification performance. Herein, the experimental results reveal that normal mangoes were efficiently distinguished from defective mangoes. Importantly, the proposed computer vision system can be used in export marketing to improve the objective evaluation of quality mangoes. It can also be used in retail stores to ensure the quality of the mangoes. To the best of our knowledge, this is the first study to propose a deep learning model for detecting mango defects. In future studies, the proposed deep learning methodology can be employed to develop a generalized computer vision system for defect identification of various fruits and vegetables.

## 7. Conclusions

This study aimed to develop a computer vision system for defect identification in mangoes using advanced machine learning techniques, which greatly benefits countries that demand enhanced export marketing of this fruit. The proposed system, which employs the CNN deep learning model, was evaluated on 800 images of mangoes and obtained a classification accuracy of 98.5%. The experimental results show that the proposed model can efficiently detect defective mangoes. This computerized system is developed to replace the manual evaluation of mango fruit, providing automated non-destructive defect detection. Therefore, the developed computer vision system is useful for the evaluators to easily detect defect in mangoes.

## Figures and Tables

**Table 1:** The proposed CNN architecture model.

Layers	Type	Number of Feature Maps	Number of Neurons in the Layer	Size of the Kernel Involves to form Each Feature Map	Stride
0	Input	3	224 × 224 × 3	-	-
1	Convolution	16	222 × 222 × 16	3 × 3 × 3	1
2	Max-pooling	16	111 × 111 × 16	2 × 2	2
3	Convolution	32	109 × 109 × 32	3 × 3 × 16	1
4	Max-pooling	32	55 × 55 × 32	2 × 2	2
5	Convolution	32	53 × 53 × 32	3 × 3 × 32	1
6	Max-pooling	32	27 × 27 × 32	2 × 2	2
7	Convolution	64	25 × 25 × 64	3 × 3 × 32	1
8	Max-pooling	64	23 × 23 × 64	3 × 3 × 64	1
9	Convolution	64	12 × 12 × 64	2 × 2	2
10	Convolution	128	10 × 10 × 128	3 × 3 × 64	1
11	Max-pooling	128	8 × 8 × 128	3 × 3 × 128	1
12	Fully Connected	-	128	-	-
13	Fully Connected	-	64	-	-
14	Output	-	2	-	-

**Table 2:** Performance measures of the proposed method.

k-Fold	Test Result	Actual		Sensitivity (%)	Specificity (%)	Accuracy (%)
		Good	Defect			
1	Good	39	1	97.5	97.5	97.5
	Defect	1	39			
2	Good	40	1	97.5	100	98.75
	Defect	0	39			
3	Good	40	1	97.5	100	98.75
	Defect	0	39			
4	Good	38	0	100	95	97.5
	Defect	2	40			
5	Good	40	0	100	100	100
	Defect	0	40			
6	Good	40	1	97.5	100	98.75
	Defect	0	39			
7	Good	40	1	97.5	100	98.75
	Defect	0	39			
8	Good	39	0	100	97.5	98.75
	Defect	1	40			
9	Good	40	1	97.5	100	98.75
	Defect	0	39			
10	Good	39	0	100	100	97.5
	Defect	0	39			

**Table 3:** Comparison of the proposed model with existing works.

References	Features	Classifier	Accuracy (%)
Proposed model		CNN	98.5
Patel et al. [19]	Morphological	Multi-linear regression models	88.75 and 97.88
Nandi et al. [10]	Color-based	Fuzzy incremental learning	87
Nandi et al. [11]	Color-based	Support vector machine	96
Huang et al. [12]	Colormetric sensor array and principal component analysis	Support vector classification	97.5
Momin et al. [16]	Geometry and shape features	Global thresholding, median filter color binarization, and morphological processing	97

## Data Availability

Data will be shared for review based on the editorial reviewer’s request.
